# Physiological ICSI (PICSI) vs. Conventional ICSI in Couples with Male Factor: A Systematic Review

**DOI:** 10.5935/1518-0557.20180027

**Published:** 2018

**Authors:** Georgina Avalos-Durán, Ana María Emilia Cañedo-Del Ángel, Juana Rivero-Murillo, Jaime Enoc Zambrano-Guerrero, Esperanza Carballo-Mondragón, Miguel Ángel Checa-Vizcaíno

**Affiliations:** 1 Laboratorio de Fertilización In-Vitro, Clínica de infertilidad y Reproducción Asistida de Toluca, IN-FIV, Metepec, Estado de México, México; 2 Departamento de Medicina Reproductiva, FILIUS, Centro de Medicina Reproductiva, San Luis Potosí, México; 3 Departamento de Medicina Reproductiva, URH, Unidad de Reproducción Humana, Quito, Ecuador; 4 Centro Nacional de Excelencia Tecnológica en Salud, Secretaria de Salud, Ciudad de México, México; 5 Laboratorio de Fertilización In-Vitro, Centro Mexicano de Fertilidad Dr. Alberto Kably, Estado de México, México; 6 Departamento de Ginecología y Obstetricia, Hospital del Mar, Hospital Universitario del Mar, Universidad Autónoma de Barcelona, Barcelona, España

**Keywords:** PICSI, physiological ICSI, hyaluronic acid, HA sperm selection, male factor

## Abstract

**Objectives:**

To determine the efficacy of the physiological ICSI technique (PICSI) vs. conventional ICSI in the prognosis of couples with male factor, with respect to the following outcome measures: live births, clinical pregnancy, implantation, embryo quality, fertilization and miscarriage rates.

**Methods:**

A systematic review of the literature, extracting raw data and performing data analysis. Patient(s): Couples with the male factor, who were subjected to *in-vitro* fertilization. Main Outcome Measures: rates of live births, clinical pregnancy, implantation, embryo quality, fertilization and miscarriage.

**Results:**

In the systematic search, we found 2,918 studies and an additional study from other sources; only two studies fulfilled the inclusion criteria for this systematic review. The rates of live births, clinical pregnancy, implantation, embryo quality, fertilization and miscarriage were similar for both groups.

**Conclusion:**

There is no statistically significant difference between PICSI vs. ICSI, for any of the outcomes analyzed in this study. Enough information is still not available to prove the efficacy of the PICSI technique over ICSI in couples with male factor.

## INTRODUCTION

 Age, associated pathologies, geographic location, consumption of alcohol, tobacco and other drugs, exposure to environmental and chemical contaminants, body and environmental temperatures, are some of the causes of male infertility (Gao *et al*., 2007; He *et al*., 2015; Imai *et al*., 2010; Verratti *et al*., 2008; Zou *et al*., 2011). Presently, the way to evaluate a semen sample is through direct spermatobioscopy, a descriptive tool that does not evaluate damage in sperm DNA (Morales *et al*., 2007; Espinoza-Navarro *et al*., 2010; World Health Organization, 2010; Cooper *et al*., 2010; González Ravina & Pacheco Castro, 2011). It is known that defects in genetic material, such as anomalies in chromatin condensation with respect to the sperm maturation process, the integrity of the DNA molecule in conjunction with the presence of DNA double chain ruptures, as well as in the single DNA chain, or the presence of chromosomal anomalies, are all related to infertility (Cortes-Gutiérrez *et al*., 2007). Oocytes are capable of repairing sperm damage, depending on the type of damage that is present in the spermatozoa (Castillo-Baso & García-Villafaña, 2012).

Diverse techniques have been developed for assisted reproduction to increase pregnancy likelihoods. One of the most used techniques is the intracytoplasmic sperm injection or conventional ICSI. However, in this technique sperm selection is subjective, since the embryologist chooses, under his/her criterion, which are the best spermatozoa seen at low resolution, thus eliminating the process of natural selection. There is a greater risk of congenital defects and miscarriages, since it is impossible to know whether the chosen spermatozoa have alterations in their nucleus or if there is DNA fragmentation (Castillo-Baso & García-Villafaña, 2012; González-Ortega *et al*., 2010).

Hence, the physiological ICSI technique arose (PICSI - physiologically selected intracytoplasmic sperm injection). This technique is based on the fact that the mature sperm head has a specific receptor that allows it to bind to hyaluronic acid (HA), the main component of the *cumulus oophorous*; this is in contrast to the immature spermatozoa, which do not have this ability to bind to HA. (Castillo-Baso & García-Villafaña, 2012; González-Ortega *et al*., 2010). It has been shown that spermatozoa that bind to HA have completed the spermatogenic process of remodeling the plasmatic membrane, cytoplasmic extrusion and nuclear maturity. Thus, they have a whole DNA and low frequency of aneuploidies and miscarriages. In this way, the genomic contribution of the spermatozoa to the zygotes can be compared to that of the spermatozoa that are selected by the *cumulus oophorous* during natural fertilization (Castillo-Baso & García-Villafaña, 2012; González-Ortega *et al*., 2010). PICSI has previously shown satisfactory results in diverse study groups, where the male factor was present and where the influence of sperm DNA fragmentation on reproduction techniques has been described (Castillo-Baso & García-Villafaña, 2012; Gongora-Rodriguez & Fontanilla-Ramirez, 2010; Parmegiani *et al*., 2010a; Majumdar & Majumdar, 2013). Nevertheless, they suggest doing further studies to this respect.

The objective of this systematic review is to determine the efficacy of the PICSI technique vs. the ICSI in the prognosis of couples with male factor, with respect to the following outcome measures: live births, clinical pregnancy, implantation, embryo quality, fertilization and miscarriage rates.

## MATERIALS AND METHODS

### Inclusion Criteria

#### Type of studies

A controlled search for clinical trials was carried out, in English and Spanish, up to August of 2015, including the following MeSH terminology: "male infertility"; "male factor"; "ICSI"; "PICSI"; "Physiological ICSI"; "Intracytoplasmic Sperm Injection"; "Physiological Intracytoplasmic Sperm Injection"; "hyaluronic acid"; "HA sperm selection".

#### Type of participants

Couples with the male factor, comparing PICSI vs. ICSI. Studies which did not fulfill the inclusion criteria were taken off the search.

#### Type of intervention

The intervention of interest for this study is the systematic review of the literature, extraction of raw data and data analyses.

#### Outcome measures

The primary outcomes of this systematic review were: miscarriage, live births and clinical pregnancy. Secondary outcomes were: implantation, embryo quality and fertilization.

### Search methods and selection of studies

#### Electronic search

The PICO (Santos *et al*., 2007) method was used to construct the research question and the bibliographic search. We performed a thorough literature search in PubMed, LILACS, Medigraphic, ELSEVIER and Cochrane. The upper time limit for the searches was August, 2015.

#### Search of other sources

The bibliographies of the included articles were searched, looking for additional references, and we contacted the main authors of the included trials, in order to solve questions and complete missing information.

### Data collection and analysis

The systematic review was carried out according to recommendations from the Cochrane Collaboration (Higgins & Green, 2011). The Review Manager 5.3 (The Cochrane Collaboration, 2014) was used to do the analyses.

#### Study selection

In an independent manner, two authors read the publications which were found through the systematized search, in order to find the trials that fulfilled the inclusion criteria of this review. We put together a list of the excluded trials, together with reasons for exclusion. Disagreements were resolved through discussion and were arbitrated by a third and fourth author of the review, when needed. Missing information was requested from the original authors, when needed.

Manual searches were carried out for abstracts of the papers found, for their possible inclusion in the review. We rejected papers that were not a report of a prospective clinical trial, if they were not about couples with male factor and compared PICSI vs. ICSI, and if they did not present quantitative outcomes with respect to live births, clinical pregnancy, implantation, embryo quality, fertilization and miscarriage rates.

#### Data extraction and management

Two reviewers, working independently, extracted data from each study, including them in an Excel sheet (search engine, title, authors, journal, years, system - SpermSlow, PICSI dish, others), designation of study (retrospective, prospective, prospective-randomized, other), type of study (abstract, full-text, other), type of intervention, inclusion criteria, exclusion criteria, objectives, results and results obtained from contact with authors). Differences in opinions were discussed and, when needed, a third person was consulted before the arbitrated consensus. In the case of missing data, or when there was a need for clarification, the study's authors were contacted.

#### Evaluation of the risk of bias in the included studies

The risk of bias in the included studies was evaluated, using the Cochrane Risk of Bias Tool (Higgins & Green, 2011); this was done independently by two of the authors. Any disagreement was resolved through discussion among the review authors, until consensus was reached. If the information was not available in the published document, we contacted one of the authors, in order to properly evaluate the trials.

We evaluated the following types of biases: random sequence generation (selection bias), allocation sequence concealment (selection bias), blinding of participants and personnel (performance bias), blinding of outcome assessment (detection bias), incomplete outcome data (attrition bias), selective outcome reporting (reporting bias) and other potential sources of bias. For the final risk of bias evaluation, we assigned values to "low risk of bias", "high risk of bias" or "uncertain risk of bias."

### Analysis

#### Measuring treatment effects

In order to show the characteristics of the studies, including their results, the information is described from the quantitative point of view, in order to combine the results of the included studies; this was done as long as they had similar characteristics as a function of the outcome variable. The values described in each study were taken into account, just as they were reported by the authors of the original studies. In the case of missing information, we contacted the authors.

The following events were estimated, using odds ratio (OR) as a measurement of treatment effect, with its respective CI of 95%: live birth, clinical pregnancy, implantation, embryo quality, fertilization and miscarriage rates. The statistical significance was established at a *p*<0.05 value. Outcome data were grouped for each study, using the Mantel-Haenszel (M-H) model and the randomized model.

The statistical heterogeneity was quantified using the statistical *I^2^* package, which shows the variation proportion among the studies, with respect to total variation, that is, the proportion of the total variation that is attributable to heterogeneity (Higgins *et al*., 2003). To estimate the variance between the studies, we used the statistical *Tau^2^* package (Higgins & Green, 2011).

All the data was analyzed using the Review Manager, version 5.3 statistical package (The Cochrane Collaboration, 2014), recommended by the Cochrane Collaboration (Higgins & Green, 2011). We reported our results according to the Guidelines for the Publication of Systematic Reviews and Meta-Analyses of Studies that Evaluate Health Interventions (*Directrices para la Publicación de Revisiones Sistemáticas y Metaanálisis de Estudios que Evalúan Intervenciones Sanitarias - PRISMA*) (Liberati *et al*., 2009).

## RESULTS

### Description of the studies

#### Search results

**Figure 1:**
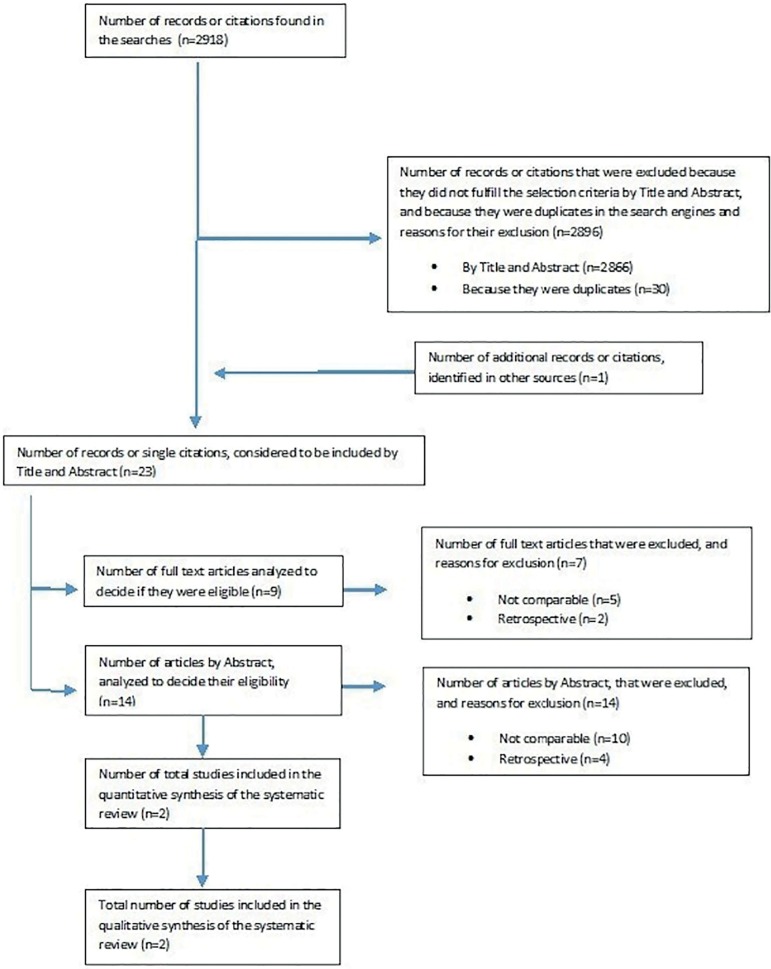
Flow diagram illustrating the selection of trials included in the meta-analysis

A total of 2,918 studies were found using all search engines (PubMed, LILACS, Medigraphic, ELSEVIER and Cochrane) and an additional study was found through other sources, up to August, 2015. From these, two studies were included in this systematic review (Castillo-Baso & García-Villafaña, 2012 ; Parmegiani *et al*., 2010a) (Figure 1).

#### Included studies

All studies that fulfilled the inclusion criteria were included. Two publications were included (Castillo-Baso & García-Villafaña, 2012; Parmegiani *et al*., 2010a). The types of PICSI systems used by these authors were SpermSlow (Parmegiani *et al*., 2010a) and PICSI dish (Castillo-Baso & García-Villafaña, 2012).

From these two studies, data pertaining to a total of 340 women was analyzed. A total of 366 treatments or cycles were performed; of these, 364 cycles involved transfers (182 were performed using the PICSI technique, where all cycles involved transfers, and 184 using the ICSI technique, where only 182 cycles involved transfers).

Although practically the same objective was analyzed in both included studies (Castillo-Baso & García-Villafaña, 2012; Parmegiani *et al*., 2010a), Parmegiani *et al*. (2010a) divided their primary objective into three secondary objectives, of which only the last one was interesting to us, and this was the one that was taken into account.

Also, these authors (Parmegiani *et al*., 2010a) did not take into account sperm morphology; however, they considered these samples as having male factor due to the total number of spermatozoa and their motility. With respect to female patients, all were candidates for ICSI, with their own oocytes and fresh cycles (Castillo-Baso & García-Villafaña, 2012).

In the Castillo-Baso & Garcia-Villafaña (2012) study, only sperm morphology was taken into account. For our study's objectives, we only used, within the three described groups of sperm morphology (≤1, 2-4% and >4% with KRUGER), the ≤1 and 2-4%, morphology groups, considering them to bear the male factor. With respect to female patients, all were candidates for ICSI, with their own oocytes and fresh cycles.

#### Excluded studies

All studies which did not fulfill the inclusion criteria were excluded. Twenty one publications were excluded for reasons shown in Table 1.

**Table 1:** Excluded studies and reasons for exclusion.

Author	Reason for exclusion from study
Azevedo *et al*., 2013	Retrospective study. It is only an abstract. Does not have complete information that is needed for its analysis. Does not analyze live birth outcomes.
Barak *et al*., 2001	It is only an abstract. It does not have the complete information needed for its analysis. It does not analyze live birth, embryo quality and miscarriage rates.
Brassesco-Macazzaga *et al*., 2009	It does not analyze the live birth and miscarriage outcomes.
Castillo-Baso *et al*., 2011	It is only an abstract. It does not have the complete information needed for its analysis. It does not analyze the live birth and miscarriage outcomes.
Hambiliki & Bungum, 2012	It is only an abstract. It does not have the complete information needed for analysis. Does not analyze live birth, implantation and miscarriage outcomes.
Lee *et al*., 2013	Retrospective study. Only an abstract. It does not have the complete information needed for analyses. Does not analyze live birth outcomes.
Majumdar & Majumdar, 2013	Patients with inexplicable infertility and normozoospermic males
Menezo *et al*., 2010	It is only an abstract. It does not have the complete information needed for the analysis. Does not analyze outcomes related to live birth, fertilization and miscarriage rates.
Parmegiani *et al*., 2010b	Retrospective study
Parmegiani *et al*., 2012	Compares two physiologic-ICSI systems: SpermSiow vs. PICSI
Santibáñez-Morales *et al*., 2012	Retrospective study. It does not analyze live birth, fertilization and miscarriage outcomes.
Saymé *et al*., 2013	Retrospective study. It is only an abstract, not having the complete information needed for analysis. It does not analyze live birth outcomes.
Van Den Bergh *et al*., 2009	It does not analyze live birth, implantation, embryo quality, clinical pregnancy and miscarriage outcomes.
Worrilow *et al*., 2005	It is only an abstract. It does not have the complete information needed for analysis. It does not analyze live birth and miscarriage outcomes.
Worrilow *et al*., 2006	Only an abstract. It does not have the complete information needed for analysis. It does not analyze live birth outcomes.
Worrilow *et al*., 2007	Is only an abstract. It does not have the complete information needed for the analysis. It does not analyze live birth outcomes
Worrilow *et al*., 2009	It does not analyze live birth, fertilization and miscarriage outcomes.
Worrilow *et al*., 2010	It does not analyze live birth and miscarriage outcomes.
Worrilow *et al*., 2011a	It Does not analyze live birth, fertilization and miscarriage outcomes.
Worrilow *et al*., 2011b	It does not analyze live birth and fertilization outcomes.
Worrilow *et al*., 2013	It does not analyze live birth and embryo quality outcomes.

### Risks of bias in the included studies

**Figure 2:**
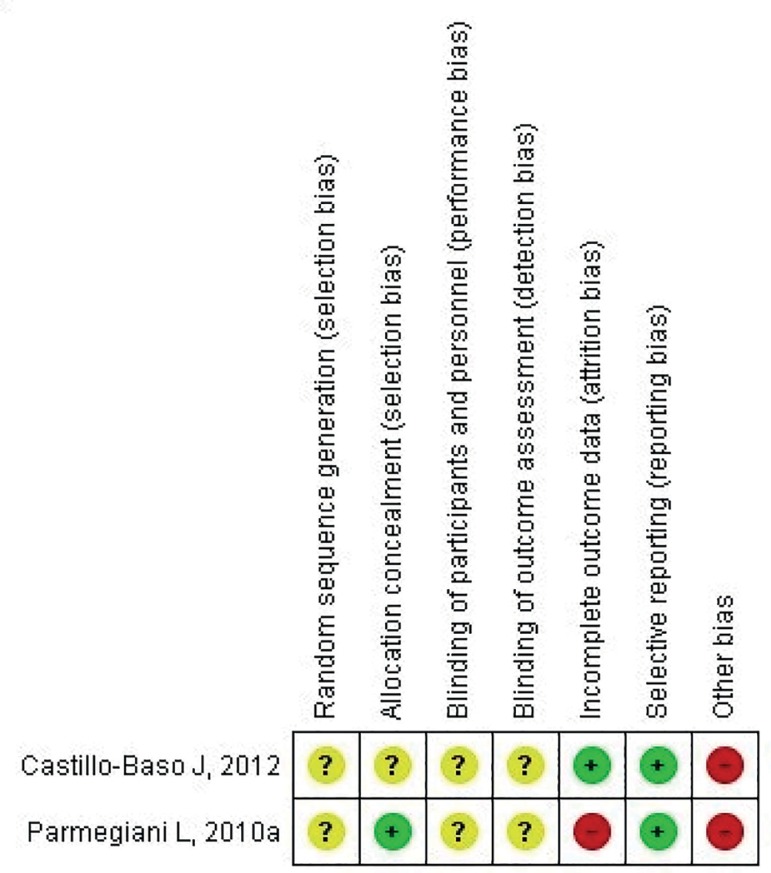
Risks of bias of the included studies

In Figure 2 we can see the different types of biases which may be present in the two papers included in this review; they are "risk of uncertain bias" (yellow: "'?" mark), "risk of low bias" (green: "+" sign) or "risk of high bias" (red: "-" sign). The "risk of uncertain bias" category is the most frequent. It is not considered to be a sign of bad quality of the included studies, given their nature. For the risk of random sequence generation (selection bias) or allocation sequence concealment (selection bias), only one study mentions being random, without mentioning the generation of the sequence and the fact that sealed envelopes were used, which were provided by a third party (Parmegiani *et al*., 2010a). The "risk of uncertain bias" was considered for both included studies (Castillo-Baso & García-Villafaña, 2012; Parmegiani *et al*., 2010a).

With respect to the risk of blinding participants and personnel (performance bias) and blinding the outcome assessment (detection bias), none of the studies (Castillo-Baso & García-Villafaña, 2012; Parmegiani *et al*., 2010a) mentions blinding of the laboratory and medical personnel, as well as of the analysts of the results; we considered this to be "risk of uncertain bias". Thus, for the risk of incomplete outcome data (attrition bias), only one study shows it in its results, desertion of two patients, not mentioning their reasons - this was considered to be a "high risk bias" (Parmegiani *et al*., 2010a). For the second study (Castillo-Baso & García-Villafaña, 2012), all patients who started the treatment, finished it; this was considered as "a low risk bias". With respect to the selective outcome reporting bias (reporting bias), in both studies (Castillo-Baso & García-Villafaña, 2012; Parmegiani *et al*., 2010a) their objective was clear and they mention at the end whether or not it was reached; this was estimated as being "low risk of bias".

Finally, for other potential sources of bias, both studies bear "high risk biases" (Castillo-Baso & García-Villafaña, 2012; Parmegiani *et al*., 2010a), since one of them does not mention, within the described semen parameters, the patients' sperm morphology (Parmegiani *et al*., 2010a). In the case of the second study, that one does not mention sperm concentration and motility (Castillo-Baso & García-Villafaña, 2012).

### Effects of interventions

**Figure 3:**
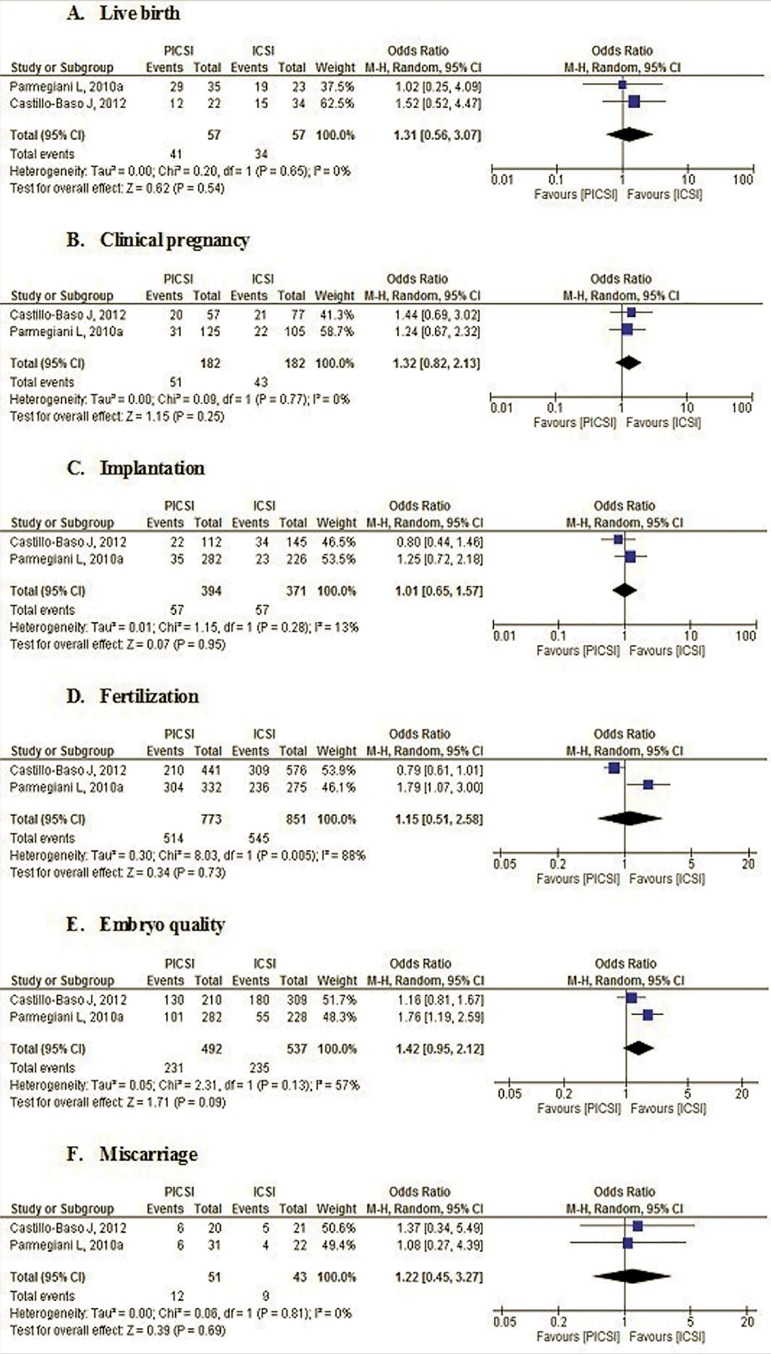
Effects of interventions

This systematic review shows that there is no statistically significant difference between both techniques, for none of the analyzed outcomes (Figure 3).

For the outcome pertaining to live births, the results of the two studies (114 events) showed no significant difference between both techniques (OR=1.31, CI 95% 0.56-3.07, *p*=0.54). The *I^2^* value was 0%, which shows "excellent statistical homogeneity".

For the clinical pregnancy outcome, results from both studies (364 events) showed no significant difference between the two techniques (OR=1.32, CI 95% 0.82-2.13, *p*=0.25). The *I^2^* value was 0%, which shows "excellent statistical homogeneity".

With respect to the implantation outcome, the results of the two studies (765 events) showed no significant difference between both techniques (OR=1.01, CI 95% 0.65-1.57, *p*=0.95). The *I^2^* value was 13%, which shows "low statistical heterogeneity".

For the fertilization outcome, the results of the two studies (1624 events), showed no significant difference between both techniques (OR=1.15, CI 95% 0.51-2.58, *p*=0.73). The *I^2^* value was 88%, which shows "high statistical heterogeneity".

For the embryo quality outcome, the results of the two studies (1029 events) showed no significant difference between both techniques (OR=1.42, CI 95% 0.95-2.12, *p*=0.09). The *I^2^* value was 57%, which indicates "moderate statistical heterogeneity".

With respect to the miscarriage outcome, the results of the two studies (94 events) showed no significant difference between PICSI vs. ICSI (OR=1.22, CI 95% 0.45-3.27, *p*=0.69). The *I^2^* value was 0%, which shows "excellent statistical homogeneity".

## DISCUSSION

The results of this systematic review were not statistically significant for all outcome measures. With respect to the risk of bias in the included studies, most of our results showed "uncertain risk of bias", since the randomization and blinding of participants is not essential, due to the nature of the studies. This risk was considered to be irrelevant, since in order to carry out the procedures, one must know which technique to apply and which one is adequate for each patient, and for this reason, we need to know the characteristics of the case. However, for the risk caused by other potential bias sources, both studies had a "high risk of bias," since they did not take into account sperm morphology, concentration and motility, which are fundamental parameters to determine the implementation of the PICSI or ICSI technique.

With respect to "statistical heterogeneity", we know that it only quantifies the variability between the study's results, and that it can be due to real differences related to the approach and execution of the included studies, or to other causes. In other words, it tries to quantify the variability in the results, that is measured in the different studies, with respect to the average global outcome, and to determine whether this variability is higher than what would be expected merely by chance.

The negative values of the statistical *I^2^* are made to be equal to zero, so that the *I^2^* is between 0% and 100%. A value of 0% shows that there is no observed heterogeneity and the greater values show a growing heterogeneity. Having markers that indicate the degrees of heterogeneity, 25% is considered to be "low statistical heterogeneity", 50% shows "moderate statistical heterogeneity" and 75% implies "high statistical heterogeneity". These markers are attributable to the statistical heterogeneity of the studies, and not to chance (Higgins *et al*., 2003). An *I^2^* of 0% is considered to have "excellent statistical homogeneity" and if variability existed in the estimation of the effects, this would be due to sampling error in the trials, and not to heterogeneity. This is the case in outcomes of live births, clinical pregnancy and miscarriage, in our review, since the results do not vary more than what would be expected from influence by chance. Finding a "low statistical heterogeneity" for the implantation outcome leads us to consider that there is scarce variability attributable to statistical heterogeneity between the studies and not to chance. Also, for embryo quality outcomes, we found "moderate statistical heterogeneity", considering it to be a moderate variability, attributable to the statistical heterogeneity between the studies and not to chance. On the other hand, for the fertilization outcome, we found a "high statistical heterogeneity", showing that the greatest part of the variability between the studies is due to heterogeneity, more than chance.

In order to decrease the "statistical heterogeneity" in this systematic review, it is important to guarantee that there is no "clinical heterogeneity" that would make the combination of results impossible, but it is not possible to maintain a "low clinical heterogeneity" because few studies fulfilled our inclusion criteria; due to their high risk of bias, resulting in a limitation. As a consequence, only two studies were included in this systematic review.

Our results do not show a statistically significant difference when comparing PICSI vs. ICSI, and these results coincide with those from Majumdar & Majumdar, 2013; Hambiliki & Bungum, 2012; Worrilow *et al*., 2010. On the other hand, a statistically significant difference favors PICSI in the study by Worrilow *et al.* (2005; 2006; 2007; 2011a, 2011b; 2013). Over the years, these authors have studied the differences between these two techniques, with respect to diverse outcomes; there are variations between the studies concerning the following outcomes: fertilization, clinical pregnancy, implantation, miscarriage and embryo fragmentation. This coincides with Azevedo *et al*. (2013) and Lee *et al*. (2013), who also found statistically significant differences when comparing PICSI vs. ICSI with respect to the miscarriage outcome, and to implantation and clinical pregnancy in the case of the study by Lee *et al*. (2013).

As far as we know, this is the first systematic review that compares PICSI vs. ICSI in the prognosis of couples with male factor, taking into account the following outcome measures: live births, clinical pregnancy, implantation, embryo quality, fertilization and miscarriage. We suggest that future studies be carried out according to the CONSORT guidelines; however, due to the nature of the intervention, it would be difficult to achieve blinding of the embryologist when performing the fertilization technique (PICSI vs. ICSI). The risk of bias could be reduced in blinding for outcome analysis and of the personnel performing the embryo transfers. It is important that these future studies provide quantitative information on results and that the rates of miscarriages, live births and clinical pregnancy be considered as primary results, without ignoring rates of implantation, fertilization and embryo quality, for the comparison of the techniques. We also recommend including the analysis of subgroups, in order to eliminate variables that affect results, such as sperm quality (morphology, concentration and motility), cause of female and male infertility, number and quality of transferred embryos, day of embryo transfer, fresh or frozen transfer, own oocyte or donated oocyte.

## CONCLUSIONS

This systematic review showed no statistically significant difference between the PICSI and the ICSI techniques, for any of the studied outcome measures: live births, clinical pregnancy, implantation, embryo quality, fertilization and miscarriage rates.

Perhaps due to the small number of clinical studies included in this review, since few studies fulfilled our inclusion criteria, due to the high risk of bias of these, it was not possible to prove the statistical efficacy of the PICSI technique over the ICSI, in couples with male factor, with respect to the studied outcome measures.

More clinical studies are needed, in accordance with the CONSORT guidelines to reduce bias risks.
